# The Piezocatalytic Degradation of Sulfadiazine by Lanthanum-Doped Barium Titanate

**DOI:** 10.3390/molecules29081719

**Published:** 2024-04-10

**Authors:** Daijun Meng, Yuqi Xiang, Ziwei Yang, Hao Yuan, Liang Tang, Shiyang Li

**Affiliations:** Key Laboratory of Organic Compound Pollution Control Engineering (MOE), School of Environmental and Chemical Engineering, Shanghai University, Shanghai 200444, Chinatang1liang@shu.edu.cn (L.T.)

**Keywords:** piezoelectric catalysis, advanced oxidation progress, antibiotics degradation

## Abstract

Piezocatalysis, a heterogeneous catalytic technique, leverages the periodic electric field changes generated by piezoelectric materials under external forces to drive carriers for the advanced oxidation of organic pollutants. Antibiotics, as emerging trace organic pollutants in water sources, pose a potential threat to animals and drinking water safety. Thus, piezoelectric catalysis can be used to degrade trace organic pollutants in water. In this work, BaTiO_3_ and La-doped BaTiO_3_ were synthesized using an improved sol–gel–hydrothermal method and used as piezocatalytic materials to degrade sulfadiazine (SDZ) with ultrasound activation. High-crystallinity products with nano cubic and spherical morphologies were successfully synthesized. An initial concentration of SDZ ranging from 1 to 10 mg/L, a catalysis dosage range from 1 to 2.5 mg/mL, pH, and the background ions in the water were considered as influencing factors and tested. The reaction rate constant was 0.0378 min^−1^ under the optimum working conditions, and the degradation efficiency achieved was 89.06% in 60 min. La-doped BaTiO_3_ had a better degradation efficiency, at 14.98% on average, compared to undoped BaTiO_3_. Further investigations into scavengers revealed a partially piezocatalytic process for the degradation of SDZ. In summary, our work provides an idea for green environmental protection in dealing with new types of environmental pollution.

## 1. Introduction

Sulfonamides, a class of broad-spectrum antibiotics, are crucial in human healthcare and more prevalently in veterinary medicine, aiding significantly in controlling bacterial infections in animals [1]. Their extensive use, however, raises environmental concerns due to their proved persistence and potential bioaccumulation [2,3], leading to antibiotic contamination in water bodies and ecosystems, primarily through the excretion of unmetabolized residues from human and animals [4]. This environmental presence, detected in natural surface water [5], urban surface water [1], wastewater [6], soil [7], and manure [8], is concerning due to its high concentrations and the consequent ecological risks, particularly in aquatic settings, where it can harm biodiversity and promote antibiotic resistance [9]. The enduring nature of sulfonamides in environments not only threatens aquatic life through its negative effects on the reproductive and growth rates of aquatic organisms [10,11] but also amplifies the public health challenge of fostered resistance genes, thereby diminishing the effectiveness of antibiotics in treating infectious diseases [12].

It was because of the escape of antibiotics from the biochemical segment of wastewater treatment facilities that researchers realized that the widely used biological methods were not effective in removing antibiotics from water bodies. However, antibiotics are not unmanageable. In response to these challenges, advanced oxidation processes (AOPs) have emerged as promising technologies for the degradation of sulfonamide antibiotics. AOPs, for example, photocatalysis [13], piezocatalysis [14], photo-piezocatalysis [15], Fenton and Fenton-like processes [16], electrochemical oxidation [17], ozonation [18], etc., have been applied to degrading antibiotics. Except AOPs, bioremediation and membrane filtration were able to partially remove antibiotics from water. Each of these methods has its advantages and limitations within specific circumstances.

Among these, piezoelectric catalysis, also known as piezocatalysis, has gained attention due to its high efficiency, environmental compatibility [19], and potential sustainability. A blueprint for sustainable piezocatalysis is to use the energy from the flow or vibration of water, which means less or even no consumption of energy in a low-entropy state. Piezocatalysis involves the generation of reactive oxygen species (ROS) under mechanical stress, offering a green and energy-efficient approach to pollutant degradation.

Many types of materials can be used as piezocatalysts if they have an asymmetric charge center while under a driving force, according to screen effect theory, and the proper band structure to generate ROS, according to band theory. With these two guidelines in mind, there are some major types of piezocatalysts that have been studied, including ZnO [20], MoS_2_ [21], perovskite [22], and some types of piezoelectric polymers [23]. Compared to other piezoelectric materials that are possible piezocatalysts, barium titanate (BaTiO_3_, BTO), a member of the perovskite family, has continuously attracted research interest owning to its high piezoelectric coefficient, excellent electrical properties, chemical and thermal stability, ease of processing and modification, and extensive research foundation.

For the processing and modification of BTO, intensive studies have been performed. In catalyst synthesis, the choice of method, parameters, and precursors is crucial, directly influencing the material’s properties and catalytic efficiency [24]. Solvothermal, solid-phase synthesis, sol–gel, and co-precipitation methods have been reported as processing methods for the piezocatalytic degradation of BTO [25,26]. Furthermore, the parameters used during the process have also been proven to significantly affect the crystal structure and consequently the catalytic efficiency [27,28,29]. As a piezocatalyst, a relatively deeper understanding of its piezocatalytic mechanism has been realized by researchers. Ongoing research explores the effectiveness of BTO in degrading diverse organic pollutants [30,31], utilizing various nanostructures [30,32] and within different working conditions [33,34,35], including the external driving force parameters, temperature, concentration, and solution condition.

A significant portion of the research indicates that enhancing the performance of piezocatalysis is not confined to a single approach; rather, it often involves combining or layering various strategies to achieve better performance improvements. The topic of elemental doping in BTO is also a critical area of study. Previous investigations have shown that doping it with isovalent or heterovalent ions at either the A or B sites may contribute to the cell parameters, subsequently enhanced piezoelectric catalytic performance [33,36,37]. Therefore, synthesizing BTO using a sol–gel–hydrothermal method, doping it with lanthanum (La) during the synthesis process, is theoretically an effective way to boost its piezocatalytic efficiency. This modification facilitates improving the charge separation and increasing the availability of its active sites for ROS generation, leading to more effective degradation of pollutants. Synthesizing BTO using the sol–gel–hydrothermal method is more green and has less of an effect on its crystallinity and morphology compared to a single hydrothermal process [26]. Also, the hydrothermal method is more convenient because sintering is not required, thereby allowing for the generation of stable nanoparticle products in large scale. 

Our study focuses on the degradation of sulfadiazine, a representative sulfonamide antibiotic. The piezocatalytic degradation of SDZ using BTO has not been studied before. By leveraging the optimized performance of lanthanum-doped BTO synthesized using the improved sol–gel–hydrothermal method, we investigate the effects of various parameters on the degradation process, including the catalyst dosage, pollutant concentration, pH levels, and the presence of interfering ions. This comprehensive approach aims to optimize the piezocatalytic conditions for the effective degradation of sulfadiazine, contributing valuable insights into the potential of piezocatalysis to mitigate the environmental impact of sulfonamide antibiotics. Through this research, we seek to develop sustainable and efficient methods for pollution control, addressing the urgent need for strategies to protect aquatic ecosystems and public health from the consequences of antibiotic contamination.

## 2. Results and Discussion

### 2.1. Characterization of BTO and BTO-La

**Figure 1:**
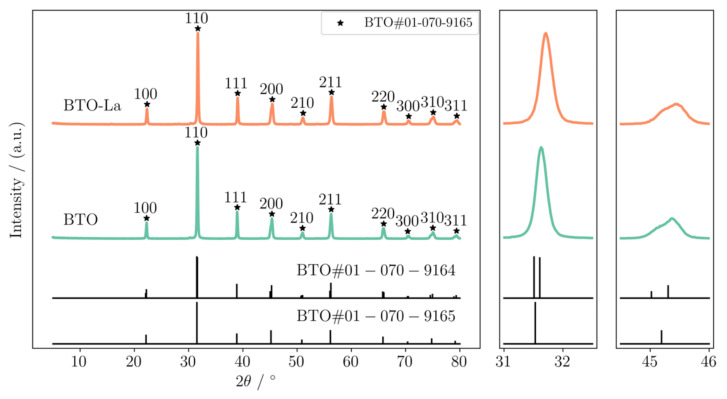
XRD patterns for BTO and BTO-La.

The XRD patterns shown in Figure 1 indicate that BTO nanoparticles were successfully synthesized, and the diffraction peaks matched the PDF cards (PDF #01-070-9165 and PDF #01-070-9164 for BTO with symmetric groups of pm3m and p4mm, respectively). In all the samples, a smaller amount of BaCO_3_ was detected. The peak shape at a diffraction angle of about 45° (2θ) indicates that there was a p4mm phase and a pm3m phase existing in the sample, corresponding to tetragonal and cubic BTO. The clear shifting of the (110) peak in the pattern of BTO-La shows smaller interplanar spacing according to Bragger’s law, which indicates a successful La-doping process. The crystallinity of the prepared materials is better than a sample synthesized using the microwave irradiation process [22] and close to that synthesized using a protracted single hydrothermal method [26].

Based on the XRD pattern, Rietveld refinement was performed to determine the detailed physical properties of the lattice structure. As calculated using the lattice parameters (a = b = 4.00192 Å and c = 4.02536 Å for undoped BTO and a = b =4.00288 Å and c = 4.02663 Å for BTO-La), the tetragonality (c/a) was slightly increased from 1.0058 to 1.0059. The c/a value is considered to be an important factor for its piezoelectric properties. The relatively higher c/a value indicates the possibility of higher piezocatalysis activity.

**Figure 2:**
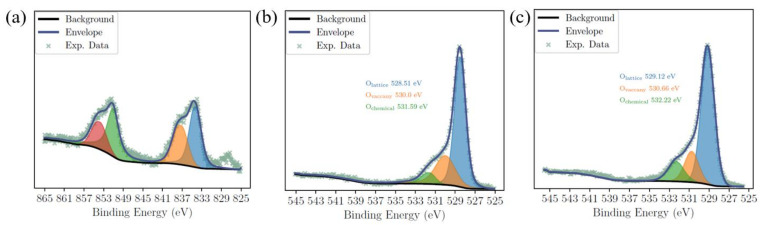
XPS scan of La 3d for BTO-La (**a**); O 1s for BTO-La (**b**); and BTO (**c**).

The XPS spectra corrected to 284.8 eV using carbon contamination are shown in the Figure 2a–c partially and in Figure S2 fully for BTO, BTO-La, and the after-reaction sample.

The La 3d spectrum shown in Figure 2a indicates the presence of La in the 3^+^ state, which shows four peaks at 834.33 eV, 837.27 eV, 851.11 eV, and 853.79 eV, while there is no significant signal detected in Figure S1c for BTO. This result indicates the successful doping of La.

The Ba 3d scan of BTO-La, illustrated in Figure S2g, shows the peaks positioned at both Ba 3d_5/2_ and Ba 3d_3/2_ can be deconvoluted into two single peaks (denoted as Ba(I) and Ba(II) for a low and high binding energy, respectively). The Ba(II) peaks were considered indicative of the surface type of BaTiO_3_, and it has been proven that this is related to the annealing process [38,39]. However, further investigation is still needed to understand the influence of this phenomenon on the piezocatalytic performance. Figure S2e shows Ti^4+^ with peaks centered at 457.33 eV and 463.05 eV with respect to Ti 2p_3/2_ and Ti 2p_1/2_, which fits well with the standard TiO_2_ spectrum, with a splitting distance of 5.70 eV.

The O 1s scan illustrated in Figure 2b shows three peaks representing perovskite lattice oxygen (O_L_), oxygen vacancies (O_V_), and chemically absorbed oxygen and barium carbonate (O_C_) at 528.51 eV, 530.00 eV, and 531.59 eV for BTO-La. Meanwhile, the peaks illustrated in Figure 2c represent BTO sites at 529.13 eV, 530.76 eV, and 532.32 eV. The O_v_/O_L_ ratio is often used to measure the concentration of OV in a material [40]. For BTO-La and BTO, the O_v_/O_L_ ratios were 0.457 and 0.240, respectively. Considering the complexity of XPS, this result implies that there are certain concentrations of OV in the BTO prepared using the sol–gel–hydrothermal method and that La doping would lead to an increase in the OV concentration.

**Figure 3:**
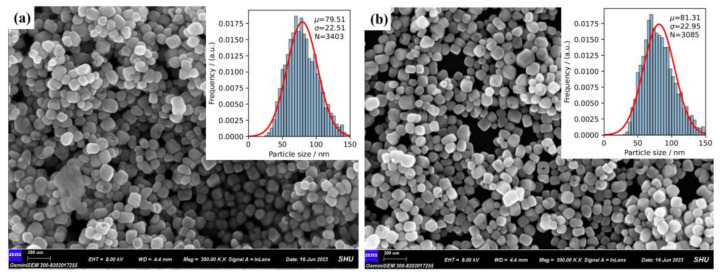
SEM images of prepared BTO (**a**) and BTO-La (**b**); the inset in images is particle size distribution (histogram in the insets) with normal distribution fitting (red line in the insets) of corresponding sample.

The SEM images show that both BTO and BTO-La have cubic and spherical nanoparticle shapes and the typical core–shell structure which is induced by the hydrothermal process. Comparison between these two images indicates no significant influence on their morphology. The samples prepared using the sol–gel–hydrothermal method have a similar morphology to a sample prepared using a single hydrothermal method [41]. The insets in Figure 3 are the average particle size distributions, which show that BTO-La has a larger and more concentrated size distribution with an average size of 81.31 nm, while it is 79.51 nm for BTO. However, the N_2_ adsorption–desorption isotherms and corresponding pore volume results shown in Figure S4 with a fitted surface area for BTO and BTO-La are 10.45 m^2^/g and 12.23 m^2^/g, respectively. This result is close to that of Lan et al. [42] but lower than that of Lv et al. [31]. It can be attributed to the much bigger particle size of the prepared samples in this study, which was induced by long-term growth using the hydrothermal method and the agglomeration of the catalyst. The obtained isotherms of both samples correspond to a type-III isothermal in accordance with the IUPAC classification, indicating weaker absorption between the catalysts and the molecules.

**Figure 4:**
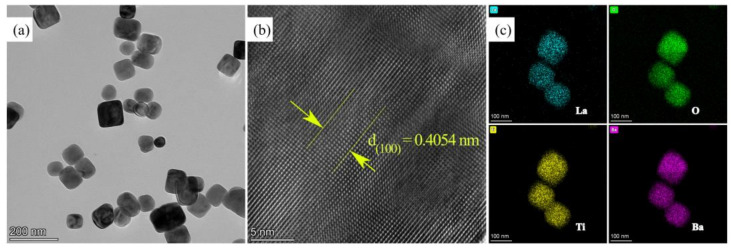
HR-TEM graphs at low (**a**) and high (**b**) magnification and EDS mapping of BTO-La (**c**).

The detailed morphologies and microstructures were probed using TEM. As shown in Figure 4a, the BTO-La exhibits similar particle sizes and cubic and irregular spherical shapes to those seen in the SEM result. The continuous orderly atomic lattice fringes focused in the core area shown in Figure 4b indicate the high crystallinity of the sample. The observations from the HR-TEM results revealed (100) crystal planes with spacings of 0.4019 nm and 0.4054 nm for BTO and BTO-La, respectively. The various interplanar spacings further substantiate the effect of heterovalent ion doping. The uniform distribution of La elements within the crystals was confirmed through EDS mapping, illustrated in Figure 4c. For Ba, Ti, O, and La, the atomic ratios are 18.66%, 17.59%, 62.55%, and 1.20%, respectively. Mott–Schottky curves and Tauc plots for BTO and BTO-La are presented in Figure S5. The results indicate that the incorporation of 5% La as a dopant does not significantly alter the band structure.

### 2.2. Effects of the Ultrasound and Recycle Tests

**Figure 5:**
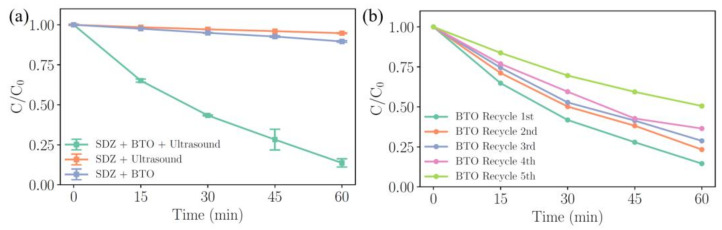
The degradation process of SDZ under various conditions as a function of the reaction time in the absence of BTO and/or ultrasound (**a**); recycle degradation process of BTO (**b**) with 1 mg/L SDZ and 1 g/L BTO dosage.

The degradation of organic pollutants through piezocatalysis has been extensively reported [30,31,33]. Controlled experiments involving piezocatalysts and external activation conditions have conclusively demonstrated that piezocatalysis can degrade organic pollutants. Figure 5a presents the experimental results with and without the presence of BTO and/or ultrasound, clearly indicating that BTO can effectively degrade SDZ under ultrasonic conditions. The TOC test results illustrated in Figure S6 show a 0.39 mg/L decrease in TOC after the reaction, close to the theoretical value calculated with the corresponding degradation efficiency (i.e., 0.42 mg/L), which means the majority of the SDZ was fully degraded by the piezocatalytic process. After five cycles of testing, presented in Figure 5b, the degradation efficiencies were 84.54%, 76.51%, 71.20%, 63.51%, and 49.38%, respectively. This decrease in the degradation efficiency may be attributed to the loss of the catalyst’s mass due to the sampling and the adsorption of by-products onto its surface. The characterization of BTO-La after five cycles through XRD, XPS, and TEM is presented in Figures S1–S3. The after-reaction XRD pattern reveals no significant alterations, indicating the stability and reusability of the modified BTO-La. Fine scans of La 3d, Ti 2p, and Ba 3d demonstrate that there are no substantial changes in the chemical states of the elements on the surface of the catalyst, and the ratio of oxygen vacancies to lattice oxygen (O_v_/O_L_) remains unchanged. The TEM observations confirm that the microstructure of the sample is preserved following the cyclic reactions. EDS mapping indicates the presence of C on the catalyst’s surface, which could be attributed to incompletely degraded SDZ. The after-reaction characterization clearly demonstrates that the surface of the catalyst involved in the AOP remained stable.

### 2.3. Effects of the Catalysis Dosage

**Figure 6:**
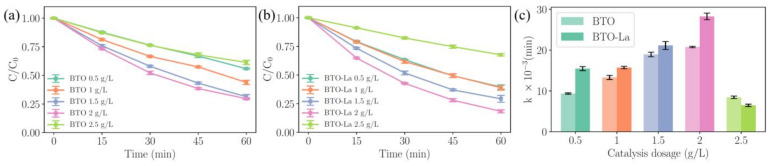
The degradation process of SDZ under various BTO dosages (**a**); BTO-La dosage (**b**) with 2.5 mg/L initial concentration of SDZ; fitting results of first-order kinetic constants (k_obs_) (**c**).

The degradation process of SDZ under various catalyst dosages is shown in Figure 5a,b for BTO and BTO-La, respectively. For the 2.5 mg/L initial SDZ concentration, the optimum dosage is 2 g/L, with a degradation efficiency of 70.36% and 81.58% for BTO and BTO-La, respectively. The apparent reaction rate constant (k_obs_, also denoted as k) was fit from the process of degradation using a first-order kinetic model. The corresponding k values for Figure 6a,b are illustrated in Figure 6c. For various BTO dosages ranging from 0.5 to 2.5 g/L, the corresponding k are 0.0094, 0.0133, 0.0190, 0.0208, and 0.0084 min^−1^. For BTO-La, the values are 0.0155, 0.0157, 0.0211, 0.0283, and 0.0065 min^−1^. It is found that BTO-La achieved an average increase of 30% in k_obs_ compared to BTO for catalyst dosages other than 2.5 g/L. This performance difference can be explained by the slightly larger c/a ratio caused by doping [20], the larger BET surface area [21], and the oxygen vacancies introduced during the doping process. The generation of oxygen vacancies in perovskite crystals due to heterovalent rare-earth doping, such as doping La into BTO, is a well-documented phenomenon [22,23,24]. Recent studies by Xiao et al. [25] and Wang et al. [26] have shown that the presence of oxygen vacancies is a crucial factor affecting the piezocatalytic performance. The mechanism is likely attributed to two competing pathways induced by doping, an increase in the carrier concentration and a decrease in the piezoelectric performance of BTO [27], however, no results have yet identified the optimal doping concentration.

Moreover, the effective reaction rate constant (k_eff_) was calculated using k_obs_, the concentration of SDZ, and the catalysis dosage. Based on the results, it is evident that except for with a catalyst dosage of 2.5 g/L, k_obs_ has a positive correlation with the catalyst dosage. However, when considering k_eff_, a negative correlation following a linear function decline is observed (Figure S1). This could be due to the agglomeration of excessive catalyst when the catalyst dosage is too high, leading to a decrease in performance [43]. When the dosage is lower, it can provide more active sites, generating more species [44].

### 2.4. Effects of the Initial Concentration of SDZ

**Figure 7:**
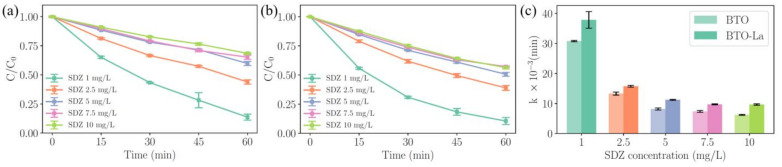
The degradation process of SDZ under various initial concentrations of SDZ for BTO (**a**) and for BTO-La (**b**) with 1 g/L catalysis dosage; fitting results of first-order kinetic constants (**c**).

The concentration of pollutants is another parameter that affects the reaction rates. The degradation process with various initial concentrations of SDZ ranging from 1 to 10 mg/L is illustrated on Figure 7a,b. During the degradation process using BTO, the corresponding k are 0.0308, 0.0133, 0.0082, 0.0074, and 0.0062 min^−1^. For BTO-La, the values are 0.0378, 0.0157, 0.0112, 0.0097, and 0.0096 min^−1^. Notably, the degradation efficiency of BTO-La achieved 89.06%, while BTO achieved 86.08%. The contrasting result shown in Figure 7c clearly indicates the effect of doping.

The results for k_obs_ and k_eff_ indicate that within the range of 1 mg/L to 10 mg/L, k_eff_ shows a power function relationship with the amount of pollutant (Figure S1), implying that the active sites are saturated and/or there is high diffusion resistance. When large quantities of pollutant molecules are present in the aqueous solution, the reactive sites may be covered by these molecules [45]. In these experiments, a difference in the reaction rates between BTO-La and BTO was also observed, with an average difference of 14.98%, further illustrating the universality of this disparity and the effectiveness of La doping in enhancing the piezocatalytic performance.

Furthermore, when presenting the experimental results on the catalyst dosage and pollutant concentration in the form of a heatmap (Figure S8), it is shown that the optimal k_obs_ and k_eff_ are achieved when the catalyst dosage is 1.0 g/L and the pollutant concentration is 1.0 mg/L.

### 2.5. Effects of the Background Conditions

**Figure 8:**
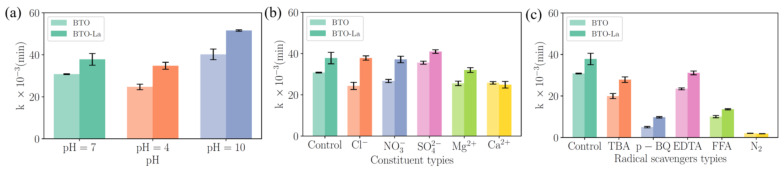
The fitting results of k_obs_ to reveal effects of various pHs of BTO and BTO-La (**a**); effects of various types of background constituents (**b**); effects of various radical scavengers for BTO and BTO-La (**c**).

The role of pH as an influencing factor in the piezocatalytic process has been mentioned recently. The overall degradation efficiency improving in alkaline conditions compared to neutral and acidic environments was observed, which is in accordance with Lv et al. [31]. The experimental results are shown on Figure 8a. Compared to under neutral conditions, the degradation efficiency increased to 95.21% and the reaction rate increased to 0.0516 min^−1^ for BTO-La. This increase was also observed for BTO, implying that the mechanism of enhancement may involve ROS reactions. This enhancement can be explained by the higher concentration of hydroxide ions adsorbed onto the surface of the catalyst due to the surface charge, affected by pH, which facilitates the generation of hydroxyl radicals, as listed in Equation (6).

Inorganic ions are ubiquitously present in aquatic environments and could potentially influence the piezocatalytic process. The effects of five common types of inorganic ions, Cl^−^, NO_3_^−^, SO_4_^2−^, Mg^2+^, and Ca^2+^, were examined at a catalyst dosage of 1 g/L and a pollutant concentration of 1 mg/L. The results illustrated on Figure 8b indicate that Cl^−^ and NO_3_^−^ do not significantly impact the performance, while SO_4_^2−^ leads to a slight increase in performance. However, Mg^2+^ and Ca^2+^ cause a certain degree of performance degradation, which may be attributed to competition for active sites due to electrostatic interactions.

### 2.6. Possible Piezocatalytic Degradation Mechanisms

To elucidate the mechanism of the piezocatalytic process, various scavengers were introduced to detect potential reactive species, and the role of oxygen throughout the reaction was verified by continuously purging the system with N_2_. The selected scavengers, along with the continuous input of N_2_, significantly inhibited the piezocatalytic process which shown on Figure 8c. Compared to the control group, TBA, p-BQ, EDTA, and FFA (corresponding to the scavenging of ·OH,··O^2−^, holes, and ^1^O_2_, respectively) resulted in decreases in the degradation efficiency of 9.19%, 44.79%, 3.81%, and 32.95%, respectively. Continuously purging the system with N_2_ almost reduced the rate to zero, underscoring the critical role of dissolved oxygen in the reaction system.

**Figure 9:**
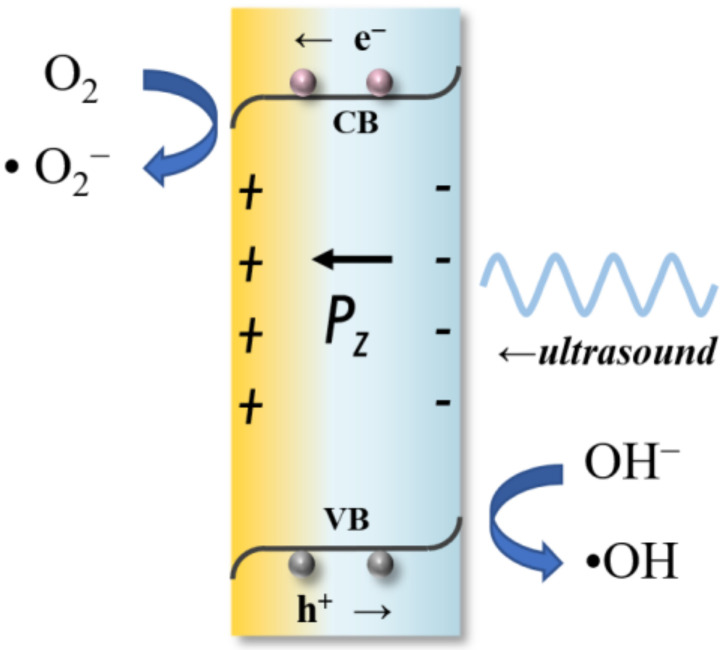
A schematic illustration of the partially piezocatalytic process for the degradation of SDZ.

Based on the above results and related reports [32,43,46], the reactions that may occur in the reaction system are as seen in the following equations and illustrated in Figure 9, where ultrasound acts as the driving force to generate ROS, such as superoxide radicals and hydroxyl radicals, to degrade organic pollutants.


(1) $$BTO\overset{\mathrm{activate(ultrasound, vibration)}}{\to }e_{\mathrm{cb}}^{-}+h_{\mathrm{vb}}^{+}$$

(2) $$O_{2}+e_{\mathrm{cb}}^{-}\to \cdot O_{2}^{-}$$

(3) $$\cdot O_{2}^{-}+H^{+}\to \cdot {\mathrm{HO}}_{2}$$

(4) $$\cdot {\mathrm{HO}}_{2}+\cdot {\mathrm{HO}}_{2}\to H_{2}O_{2}+O_{2}$$

(5) $$e_{\mathrm{cb}}^{-}+H_{2}O_{2}\to \cdot \mathrm{OH}+{\mathrm{OH}}^{-}$$

(6) $$h_{\mathrm{vb}}^{+}+{\mathrm{OH}}^{-}\to \cdot \mathrm{OH}$$

(7) $$\mathrm{Organic molecules}+\cdot \mathrm{OH}/O_{2}^{-}\to \mathrm{harmless products}$$

The mechanism of piezocatalytic degradation with BTO is thought to involve the generation of ROS under irradiation of ultrasound as a mechanical stress source. The piezoelectric potential induced by the deformation of the material promotes the separation of charge carriers, facilitating the formation of ·OH and ·O^2−^ radicals. These radicals are highly reactive and capable of breaking down complex organic molecules into simpler, non-toxic compounds [47].

Regarding the enhancement of the degradation efficiency resulting from elemental doping, investigations have been conducted into the lattice parameters, band structure, surface properties, and defect conditions in reported studies [29,46,48,49]. Consequently, the degradation efficiency should be considered an indicator influenced by a multitude of factors. In this study, UV-DRS confirmed that the modified BTO-La had no significant impurity energy level, which rules out one of common causes of performance enhancement. However, considering the results of the O 1s XPS, the BTO-La is hypothesized to enhance the piezocatalytic process by introducing additional oxygen vacancies, thereby increasing the efficiency of the degradation. In fact, this hypothesis was also mentioned by Xiao et al. [50] and Wang et al. [51]. Moreover, in more detailed studies of photocatalysis, the presence of a suitable amount of oxygen vacancy defects has been found not to weaken but rather to significantly enhance the photocurrent density, thereby boosting the associated reactive performance [52]. Therefore, speculation regarding the enhanced performance of BTO-La is reasonable, but further detailed characterization and experimental design are essential.

## 3. Conclusions

La-doped BTO nanoparticles were successfully synthesized using an improved sol–gel–hydrothermal method. The prepared BTO-La had a nano cubic and spherical morphology and exhibited higher piezocatalytic activity for SDZ degradation compared to undoped BTO, achieving an average increase of 14.98% in its degradation efficiency under various pollutant concentrations and catalyst dosages. This increase may be attributed to oxygen vacancy. Moreover, its performance remained stable after five cycles of degradation, maintaining degradation capability for SDZ across a wide range of pH conditions and in the presence of inorganic ions. Further quenching experiments indicated that superoxide and hydroxyl radicals play a significant role in the degradation process of SDZ, suggesting that La-doped BTO is a promising candidate for advanced oxidation technologies.

## 4. Materials and Methods

All the chemicals, including tetrabutyl titanate ((C_3_H_7_O)_4_Ti, ≥99%, RG), barium hydroxide octahydrate (Ba(OH)_2_•8H_2_O, 98%, AR), ethanol (≥99.8%, GR), ammonium hydroxide (25~28%, Ultra Grade), lanthanum hydroxide (La(OH)_3_, ≥99.95%, RG), sulfadiazine (SDZ, C_10_H_10_N_4_O_2_S, ≥99%, RG), acetonitrile (CH_3_CN, ≥99.9%, HPLC grade), tert-butanol (TBA, ≥99.5%, GCS), furfuryl alcohol (FFA, ≥99%, RG), and p-benzoquinone (p-BQ, RG), were purchased from Titan™ (Shanghai, China) and used without further purification. All chemicals used in this study were of analytical grade unless otherwise stated. All the experimental solutions were prepared with ultrapure water (18.2 MΩ).

### 4.1. Synthesis of BTO

The BTO was synthesized using the sol–gel–hydrothermal method. First, 0.01 M tetrabutyl titanate and 0.015 M barium hydroxide were added into 20 mL ethanol and 30 mL DI water, respectively. For the BTO-La, lanthanum hydroxide and barium hydroxide were added simultaneously, at an atomic ratio of 5%. Then, these two solutions were mixed, ammonia water was added into the solution, and then the solution was stirred for 2 h at 80 °C. After stirring, the solution was transferred into a hydrothermal synthesis reactor and heated in the oven for 16 h at 200 °C. The hydrothermal product was washed and centrifuged with ethanol, 0.1 M nitric acid, and DI water 2 times. Finally, the product was dried at 60 °C for 24 h.

### 4.2. Characterization

Scanning electron microscopy (SEM, Zeiss GeminiSEM 300, Oberkochen, Germany) was used to determine the surface morphology of the BTO. The size distribution was analyzed using OpenCV; each particle in the SEM images was separated using the Segment Anything model as the image detector. X-ray diffraction (XRD, Bruker D8, Billerica, MA, USA) with Cu Kα radiation with a voltage of 40 kV and a current of 40 mA was used to determine the crystallinity and lattice parameters of the BTO nanoparticles. X-ray photoelectron spectroscopy (XPS, Thermo Scientific K-Alpha+, Waltham, MA, USA) with Al Kα as the X-ray source was used to determine the doping results, and all the binding energies of the samples were corrected by referencing the C 1s peak to 284.8 eV. High-resolution transmission electron microscopy (HR-TEM, Thermo Fisher Talos F200S G2, Waltham, MA, USA) with an energy-dispersive X-ray spectroscopy detector (EDS) was used to determine the microstructure and the elemental distribution of the catalyst. The specific surface area was measured according to the physical adsorption of N_2_ at 77 K using an auto-adsorption system (Micromeritics ASAP 2460, Norcross, GA, USA) and calculated using the Brunauer–Emmett–Teller (BET) equation. The impedance–potential curves (Mott–Schottky plots) were generated using an electrochemical workstation (Chenhua CHI660E, Shanghai, China) to determine the impedance of and the band information for the BTO nanoparticles. The UV-Vis diffuse reflectance spectra (UV-DRS) were recorded using a spectrophotometer (Shimadzu UV2600, Kyoto, Japan) in the region of 200~800 nm.

### 4.3. Piezocatalytic Degradation Experiment

The piezocatalytic degradation experiments were carried out in the presence of BTO under ultrasound vibration. A certain amount of BTO was mixed into the solution of SDZ. After dark adsorption, a glass vessel containing 40 mL of the well-mixed solution was placed in the ultrasonic machine (YM-020PLUS, Guangzhou, China) with a frequency of 40 kHz at 120 watts. At intervals of 15 min, 1 mL of the reaction solution was withdrawn and centrifuged at 14,000 rpm for further analysis of the residual SDZ concentration. To prevent heat from being generated by the ultrasound, the water in the ultrasonic machine was replaced each interval. After each cycle during the recycle test, the after-reaction solution was centrifuged, and the dried sediment was used for the next cycle of characterizations. The pH was controlled using phosphate buffer solution in the pH effect tests and confirmed using a pH meter after the reaction. The effects of ions were tested by adding NaCl, NaNO_3_, Na_2_(SO_4_)_2_, CaCl_2_, and MgCl_2_. All the experiments were conducted in triplicate, and the mean values with standard deviation are reported and shown in the figures. In the quenching tests, and TBA, p-BQ and FFA were employed as scavengers of ·OH, ·O^2−^, and singlet oxygen (^1^O_2_), respectively.

The SDZ was analyzed using high-performance liquid chromatography (HPLC, Thermo Scientific UltiMate 3000, Sunnyvale, CA, USA) using a UV detector set at 270 nm and an Acclaim™ 120 C18 column (150 mm × 4.6 mm; 5 μm) maintained at 40 °C. DI water with 0.1% phosphoric acid and acetonitrile (60:40, *v*:*v*) with a flow rate of 1.0 mL·min^−1^ was used as the mobile phase. The total organic carbon (TOC) was measured using the TC-IC method with a TOC analyzer (Shimadzu TOC-L, Kyoto, Japan).

## Data Availability

Data are contained within the article.

## Supplementary Materials

The following supporting information can be downloaded at: https://www.mdpi.com/article/10.3390/molecules29081719/s1, Figure S1: XRD patterns of as-prepared and after-recycle-reaction of BTO-La; Figure S2: XPS survey and fine scan of as-prepared BTO (a–f), BTO-La (g–l) and after-recycle-reaction of BTO-La (m–s); Figure S3: HR-TEM graphs at low (a) and high (b) magnification, and EDS mapping of after-recycle-rection of BTO-La (c); Figure S4: Nitrogen adsorption-desorption isotherms and insets (the corresponding pore size distribution curves) of BTO (a) and BTO-La (b); Figure S5: Mott-Schottky curves (a) and Tauc plot (b) result of BTO and BTO-La. The inset in (b) is absorbance while performing UV-DRS test; Figure S6: TOC of experimental solution before and after reaction with 1 mg/L SDZ and 1 g/L BTO-La; Figure S7: Correlation between k_eff_ and catalysis dosage (a); SDZ concentration (b); Figure S8: Heatmap of k as the function of SDZ concentration and catalyst dosage (left column) and actual test point (right column); Figure S9: The degradation process of SDZ with BTO under various pH (a); background constituent (b); radical scavengers (c); with BTO-La; under various pH (c); background constituent (d); radical scavengers (f);Table S1: The k_obs_ and degradation efficiency of the tested groups in this study.
