# Supplementary material for: The Piezocatalytic Degradation of Sulfadiazine by Lanthanum-Doped Barium Titanate
Source: Molecules. 2024 Apr 10;29(8):1719. doi: 10.3390/molecules29081719 (PMC11051747; doi:10.3390/molecules29081719)
Supplement: Supplementary file 1 [file molecules-29-01719-s001.zip › molecules-2906907-supplementary.pdf]

The Piezocatalytic Degradation of Sulfadiazine by Lanthanum-Doped Barium Titanate

Daijun Meng †, Yuqi Xiang †, Ziwei Yang, Hao Yuan, Liang Tang ‡ and Shiyang Li *,‡

Key Laboratory of Organic Compound Pollution Control Engineering (MOE), School of Environmental and Chemical Engineering, Shanghai University, Shanghai 200444, China; tang1liang@shu.edu.cn (L.T.)

* Correspondence: lishiyang@shu.edu.cn; Tel./Fax: +86-21-65982592

† These authors contributed equally to this work.

‡ These authors also contributed equally to this work.

Tabel S1 The k_{obs} and degradation efficiency of the tested groups in this study

Catalysis dosage (g/L)	SDZ concentration (mg/L)	Label	Condition	k_obs_average (x10^{-3} min^{-1})	k_obs_std (x10^{-3} min^{-1})	degradation efficiency (%)
0.5	1	BTO	-	17.51	0.66	65.06%
0.5	2.5	BTO	-	9.35	0.17	44.12%
1	1	BTO	-	30.79	0.20	86.08%
1	10	BTO	-	6.20	0.16	31.50%
1	2.5	BTO	-	13.29	0.51	56.08%
1	5	BTO	-	8.16	0.34	40.17%
1	7.5	BTO	-	7.36	0.29	34.79%
1.5	2.5	BTO	-	18.94	0.56	68.60%
1.5	5	BTO	-	6.28	0.30	31.48%
2	2.5	BTO	-	20.76	0.16	70.36%
2	7.5	BTO	-	4.76	0.10	24.02%
2.5	10	BTO	-	5.64	0.21	28.42%
2.5	2.5	BTO	-	8.43	0.30	38.66%
0	1	BTO	-	0.91	0.13	5.23%
1	1	BTO	no us	1.77	0.05	10.41%
1	1	BTO	pH10	40.19	2.52	90.14%
1	1	BTO	pH4	24.68	1.34	76.55%
1	1	BTO	Cl-	24.33	1.72	76.93%
1	1	BTO	NO3-	26.71	0.79	78.48%
1	1	BTO	Mg2+	25.59	1.08	77.33%
1	1	BTO	Ca2+	25.83	0.61	80.63%
1	1	BTO	SO42-	35.59	0.73	88.65%
1	1	BTO	TBA	19.95	1.24	68.62%
1	1	BTO	p-BQ	4.99	0.34	26.02%
1	1	BTO	EDTA-2Na	23.43	0.44	75.27%
1	1	BTO	FFA	10.06	0.55	45.30%
1	1	BTO	N2	2.01	0.07	12.02%
1	1	BTO	Recycle1	30.49	-	84.54%
1	1	BTO	Recycle2	23.19	-	76.51%
1	1	BTO	Recycle3	20.45	-	71.20%
1	1	BTO	Recycle4	17.53	-	63.51%
1	1	BTO	Recycle5	11.55	-	49.38%
0.5	1	BTO-La	-	16.23	0.48	61.10%
0.5	2.5	BTO-La	-	15.47	0.50	60.36%
1	1	BTO-La	-	37.83	2.77	89.06%
1	10	BTO-La	-	9.62	0.24	43.64%
1	2.5	BTO-La	-	15.73	0.31	60.99%
1	5	BTO-La	-	11.22	0.17	49.37%
1	7.5	BTO-La	-	9.72	0.15	42.77%
1.5	2.5	BTO-La	-	21.13	0.98	70.55%
1.5	5	BTO-La	-	6.26	0.11	30.53%
2	2.5	BTO-La	-	28.26	0.81	81.58%
2	7.5	BTO-La	-	4.20	0.15	22.05%
2.5	10	BTO-La	-	5.73	0.21	28.82%

2.5	2.5	BTO-La	-	6.47	0.29	32.29%
0	1	BTO-La	-	0.73	0.08	4.39%
1	1	BTO-La	no us	4.32	0.28	23.25%
1	1	BTO-La	pH10	51.55	0.33	95.21%
1	1	BTO-La	pH4	34.77	1.67	87.07%
1	1	BTO-La	Cl-	37.87	1.05	90.33%
1	1	BTO-La	NO3-	37.18	1.56	89.91%
1	1	BTO-La	Mg2+	32.04	1.18	85.11%
1	1	BTO-La	Ca2+	24.91	1.60	76.02%
1	1	BTO-La	SO42-	40.97	0.87	90.80%
1	1	BTO-La	TBA	27.85	1.37	79.87%
1	1	BTO-La	p-BQ	9.75	0.31	44.27%
1	1	BTO-La	EDTA-2Na	31.12	0.91	85.25%
1	1	BTO-La	FFA	13.60	0.28	56.11%
1	1	BTO-La	N2	1.81	0.07	10.53%
1	1	BTO-La	Recycle1	39.27	-	90.14%
1	1	BTO-La	Recycle2	33.45	-	85.41%
1	1	BTO-La	Recycle3	29.19	-	84.92%
1	1	BTO-La	Recycle4	23.40	-	75.46%
1	1	BTO-La	Recycle5	19.83	-	72.32%

'-' in labels means under normal ultrasound activation, '-' in std means no parallel test have been done.

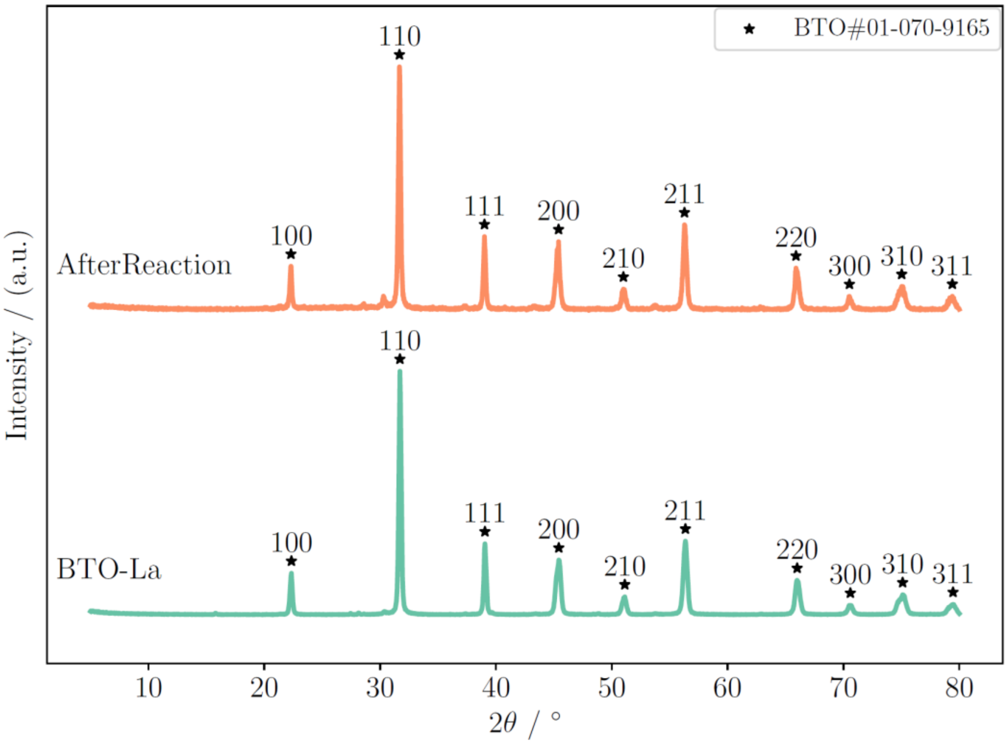

Figure S1. XRD patterns of as-prepared and after-recycle-reaction of BTO-La.

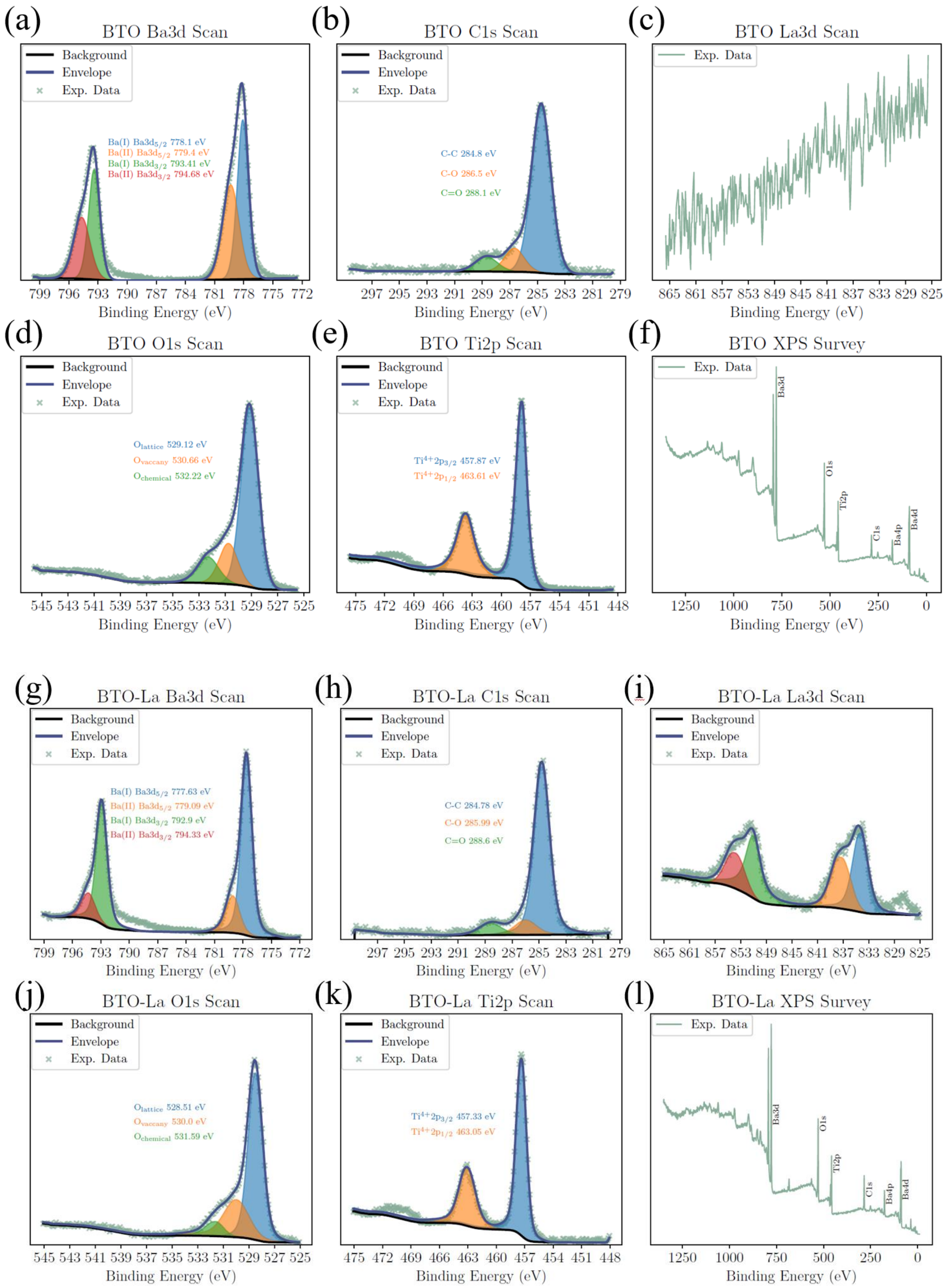
(a)
BTO Ba3d Scan
Background
Envelope
Exp. Data
Ba(I) Ba3d5/2 778.1 eV
Ba(II) Ba3d5/2 779.4 eV
Ba(I) Ba3d3/2 793.41 eV
Ba(II) Ba3d3/2 794.68 eV
Binding Energy (eV)
(b)
BTO C1s Scan
Background
Envelope
Exp. Data
C-C 284.8 eV
C-O 286.5 eV
C=O 288.1 eV
Binding Energy (eV)
(c)
BTO La3d Scan
Exp. Data
Binding Energy (eV)
(d)
BTO O1s Scan
Background
Envelope
Exp. Data
Olattice 529.12 eV
Ovaccany 530.66 eV
Ochemical 532.22 eV
Binding Energy (eV)
(e)
BTO Ti2p Scan
Background
Envelope
Exp. Data
Ti4+2p3/2 457.87 eV
Ti4+2p1/2 463.61 eV
Binding Energy (eV)
(f)
BTO XPS Survey
Exp. Data
Ba3d
O1s
Ti2p
C1s
Ba4p
Ba4d
Binding Energy (eV)
(g)
BTO-La Ba3d Scan
Background
Envelope
Exp. Data
Ba(I) Ba3d5/2 777.63 eV
Ba(II) Ba3d5/2 779.09 eV
Ba(I) Ba3d3/2 792.9 eV
Ba(II) Ba3d3/2 794.33 eV
Binding Energy (eV)
(h)
BTO-La C1s Scan
Background
Envelope
Exp. Data
C-C 284.78 eV
C-O 285.99 eV
C=O 288.6 eV
Binding Energy (eV)
(i)
BTO-La La3d Scan
Background
Envelope
Exp. Data
Binding Energy (eV)
(j)
BTO-La O1s Scan
Background
Envelope
Exp. Data
Olattice 528.51 eV
Ovaccany 530.0 eV
Ochemical 531.59 eV
Binding Energy (eV)
(k)
BTO-La Ti2p Scan
Background
Envelope
Exp. Data
Ti4+2p3/2 457.33 eV
Ti4+2p1/2 463.05 eV
Binding Energy (eV)
(l)
BTO-La XPS Survey
Exp. Data
Ba3d
O1s
Ti2p
C1s
Ba4p
Ba4d
Binding Energy (eV)

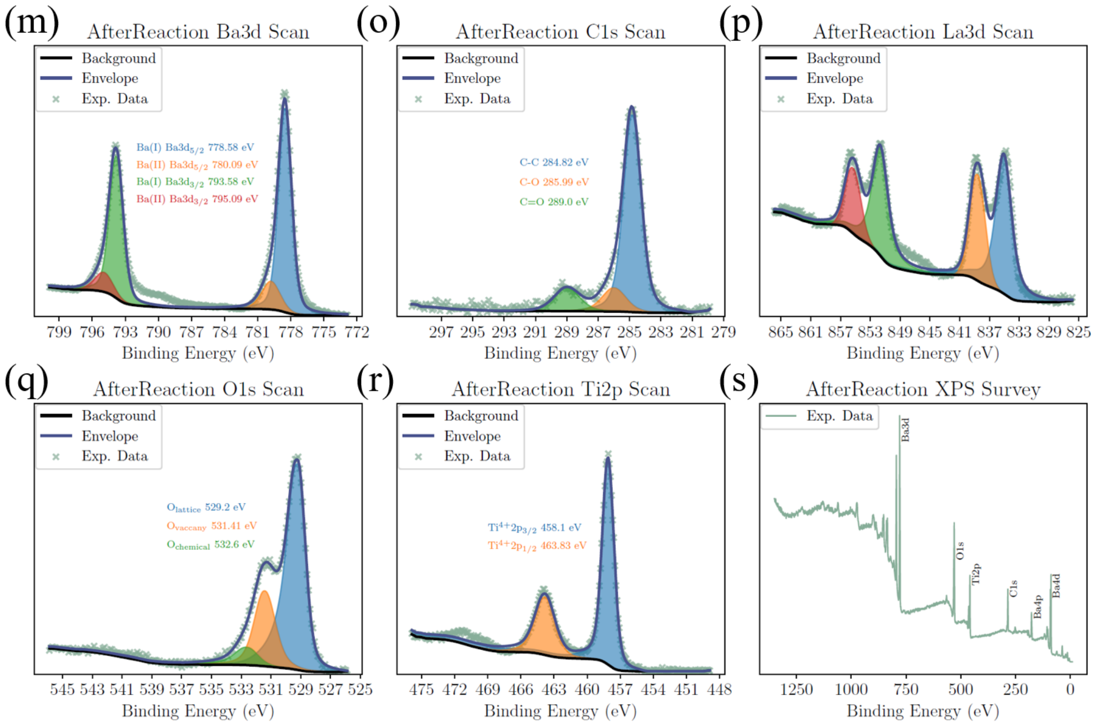

Figure S2. XPS survey and fine scan of as-prepared BTO (a-f), BTO-La(g-l) and after-recycle-reaction of BTO-La (m-s).

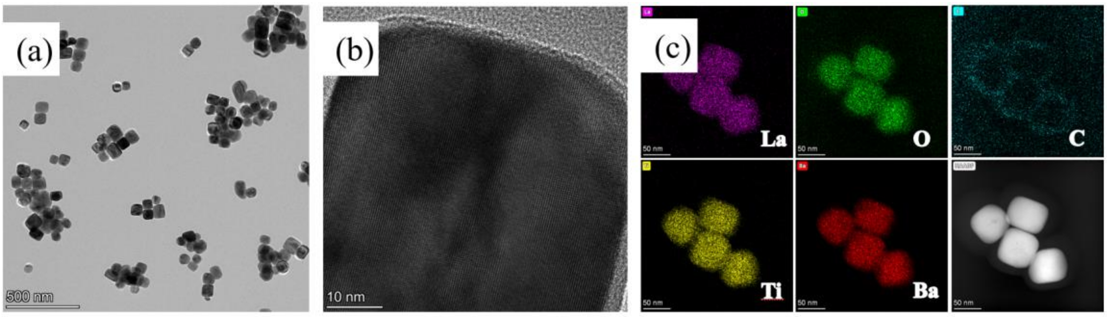

Figure S3. HR-TEM graphs at low (a) and high (b) magnification, and EDS mapping of after-recycle-rection of BTO-La (c)

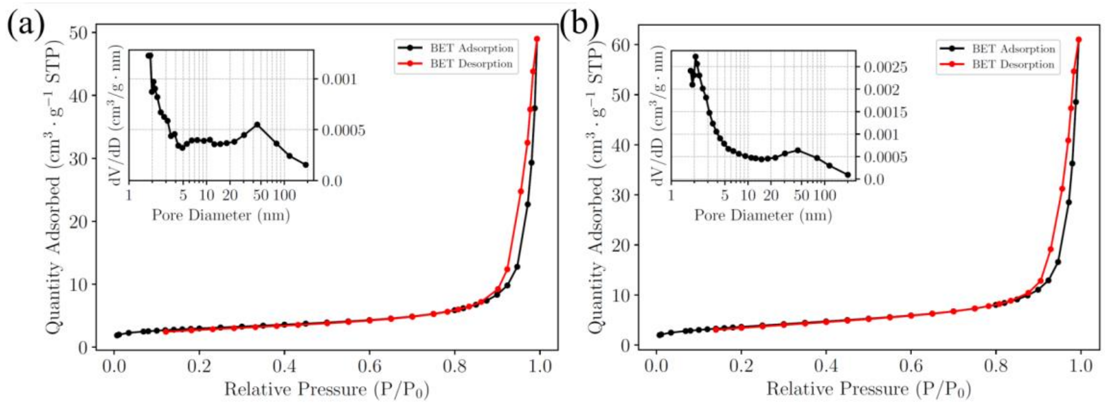

Figure S4. Nitrogen adsorption-desorption isotherms and insets (the corresponding pore size distribution curves) of BTO (a) and BTO-La (b)

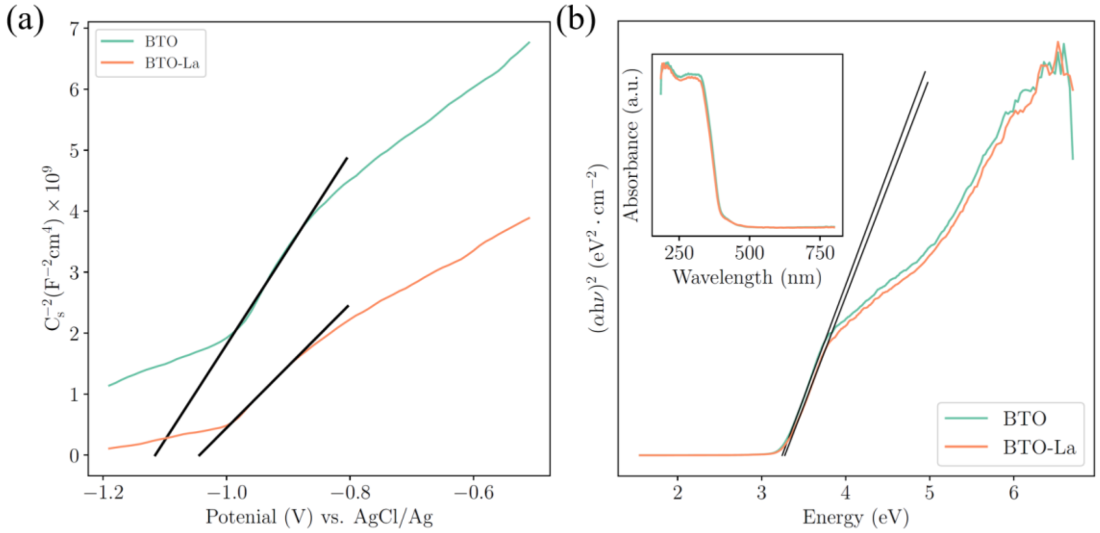

	E_v (V) vs. NHE	E_g (eV)	E_c (V) vs. NHE
BTO	-0.92	3.24	2.32
BTO-La	-0.85	3.28	2.43

Figure S5. Mott-Schottky curves (a) and Tauc plot (b) result of BTO and BTO-La. The inset in (b) is absorbance while performing UV-DRS test. Corresponding columns of table below the plots are valance position, band gap and conduction position.

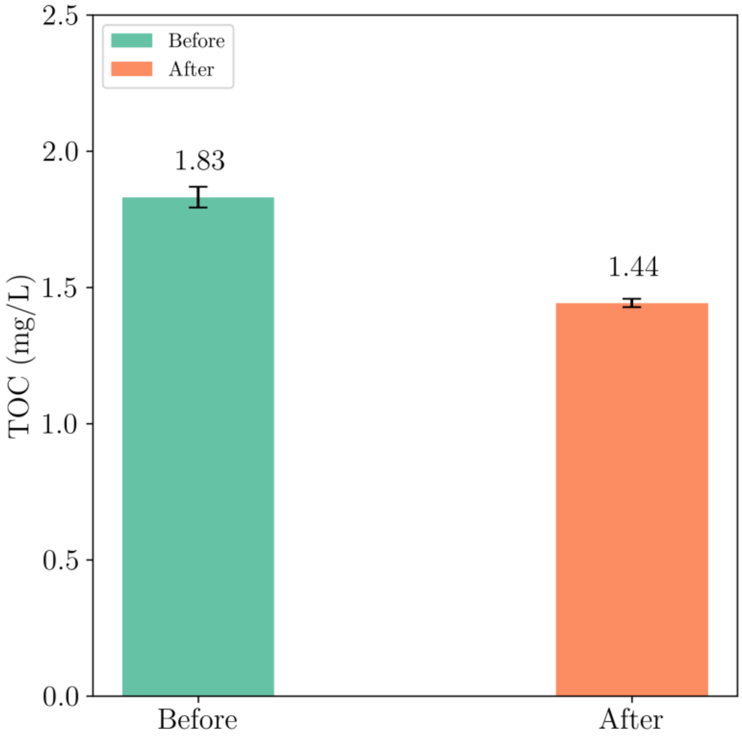

Figure S6. TOC of experimental solution before and after reaction with 1 mg/L SDZ and 1 g/L BTO-La

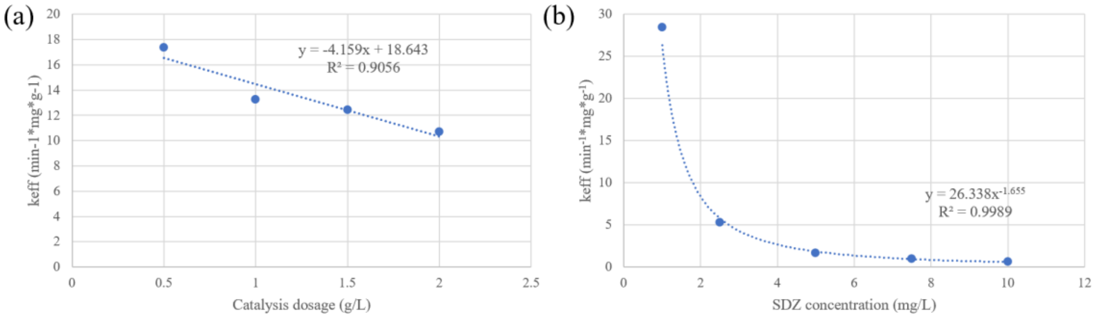

Figure S7. Correlation between k_{eff} and catalysis dosage (a); SDZ concentration (b).

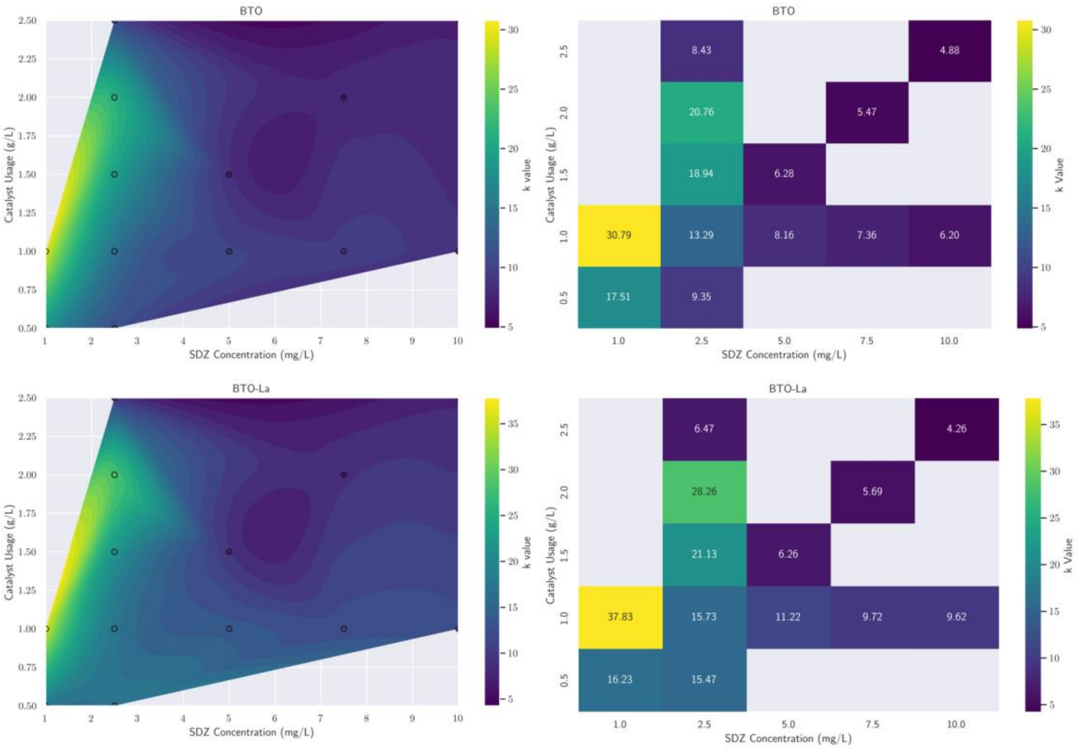

Figure S8. Heatmap of k as the function of SDZ concentration and catalyst dosage (left column) and actual test point (right column).

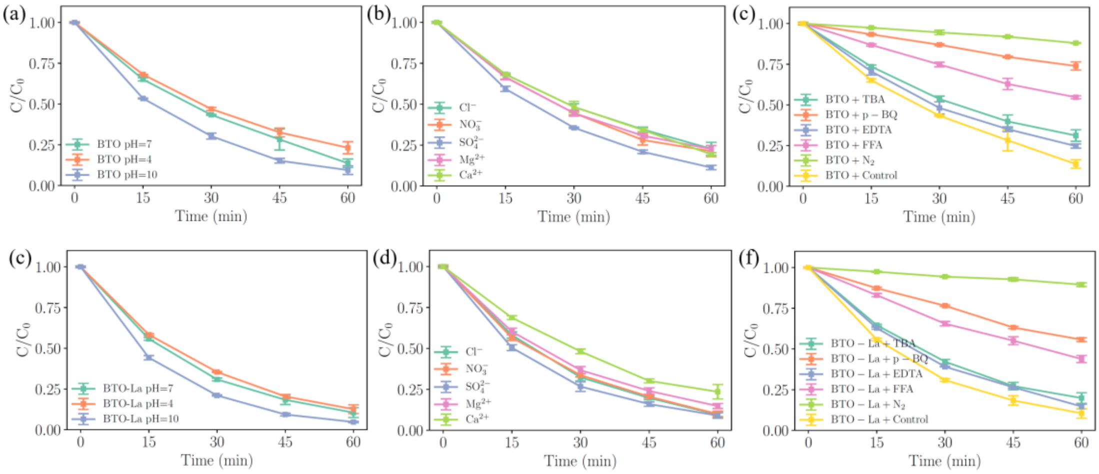

Figure S9. The degradation process of SDZ with BTO under various pH (a); background constituent (b); radical scavengers (c); with BTO-La; under various pH (c); background constituent (d); radical scavengers (f)
